# Parental bonding, depression, and suicidal ideation in medical students

**DOI:** 10.3389/fpsyg.2022.877306

**Published:** 2022-08-04

**Authors:** Stefano Tugnoli, Ilaria Casetta, Stefano Caracciolo, Jacopo Salviato

**Affiliations:** Neurological, Psychiatric, and Psychological Sciences Section, Department of Neuroscience and Rehabilitation, Faculty of Medicine, Pharmacy and Prevention, University of Ferrara, Ferrara, Italy

**Keywords:** medical students, depression, suicidal ideation, parental bonding, gender

## Abstract

**Background:**

The psychological condition of university students has been the focus of research since several years. In this population, prevalence rates of depression, suicidal ideation, anxiety disorders and substance abuse are higher than those of the general population, and medical students are more likely to have mental health issues than other students.

**Aims:**

This study deals with the psychological condition of medical students, with a focus on correlations between depression, suicidal ideation and the quality of the perceived parenting style. Gender differences were also considered.

**Methods:**

A cross-sectional study was conducted on a population of medical students, with an online questionnaire consisting of a personal data sheet for demographic and anamnestic data, and of three self-rating scales: the *Beck Depression Inventory II* (BDI-II), for the screening of depressive symptoms; the *Beck Hopelessness Scale* (BHS), to assess suicidal ideation; the *Parental Bonding Instrument* (PBI), to investigate the memory of the attitude of one’s parents in the first 16 years of life. Two main affective dimensions were considered by PBI: “care” (affection and empathy) and “protection” (intrusiveness, controlling and constraint). Four different patterns of parenting styles are so evidenced: Neglectful Parenting (low care/low protection), Affectionless Control (low care/high protection), Optimal Parenting (high care/low protection), and Affectionate Constraint (high care/high protection).

**Results:**

Overall, 671 students (182 males and 489 females) participated. Females, compared to males, experienced more distress and self-injurious behaviors, while males experienced more drugs or alcohol abuse. The BHS and BDI-II scores correlated positively with the PBI score for “protection” and negatively with that for “care.” Affectionless Control and Neglectful Parenting were associated with higher medians of BHS and BDI-II scores.

**Conclusion:**

The study confirms that the undergraduate medical student population has higher prevalence of depression and suicidal ideation than those detectable in the general population (respectively, 50.2% and 16.7% vs. 15–18% and 9.2%) and that some specific parenting styles correlate with these two clinical variables. The impact of Affectionless Control and Neglectful Parenting on suicidal ideation and depressive symptomatology was more pronounced in females than in males. For males, the role of the father seemed to have less impact on the affective roots of suicidal thoughts and depression.

## Introduction

The transition between high school and university is a crucial period in biological, psychological, social development, through the growth of new bonds, a new sense of self, and a rise in autonomy and responsibility (Taylor et al., 2014). Based on current estimates, 35% of college students met the diagnostic criteria for at least one common mental health illness or a related health issue (*WHO World Mental Health International College Student project* – Auerbach et al., 2018), with prevalence rates higher than those of the general population of depression, suicidal ideation, anxiety disorders, and substance use or abuse. The most common disorder among college students is depression (21% lifetime prevalence), followed by generalized anxiety disorder (18%–16%), panic disorder (5%) and bipolar disorder (3.5%; Auerbach et al., 2018). Suicidal ideation among university students is around 6.7%, while suicide plans and attempts are, respectively, 1.6% and 0.5% (Downs and Eisenberg, 2012). 9.5% of university students screened positive for an eating disorder (Eisenberg et al., 2011) and about 44% for binge drink; 12.5% suffer from alcohol dependence and 7.8% abuse it (although the percentages fluctuate globally); about 23% of male students and 16% of female students are current marijuana users; as a whole, drug use disorder affects about one student out of 20 (Pedrelli et al., 2015).

Admission to medical school and the period leading up to graduation are extremely competitive and demanding. Medical school students are more likely to experience mental health problems than other students (Rotenstein et al., 2016) and they are at greater risk of developing mental disorders or using illegal substances (Mousa et al., 2016; Moutinho et al., 2019).

These symptoms are associated with decreased academic performance, poor quality healthcare, and higher medical errors (Agnafors et al., 2021). Female sex, exposure to recent stressful life events, excessive smartphone use, and poor sleep quality are all risk factors for the development of mental disorders in this student population (Lemola et al., 2015).

Additionally, medical students with mental health issues seek help infrequently (Gold et al., 2015): more than half of medical students who meet the diagnostic criteria for a mental disorder are reticent to seek professional help due to the fear of getting stigmatized (Mehta and Edwards, 2018). Furthermore, after graduation, the concern of stigma, as well as financial and professional repercussions, is a substantial obstacle to seeking assistance between doctors (Pingani et al., 2016; Deb et al., 2019; Thornicroft et al., 2019).

The prevalence rate of depression is higher in undergraduate medical students than in the general population (Lim et al., 2018), and the overall prevalence of depression or depressive symptoms among medical students is 27.2% (Rotenstein et al., 2016), with the higher rates (33%) in the first-year students (Puthran et al., 2016). The prevalence of suicidal ideation, reported as having occurred over the past 2 weeks to the past 12 months, was 11.1% (Rotenstein et al., 2016). The assessment of hopelessness among university students, through the use of the Beck Hopelessness Scale, detect an average value of 3.26 with a range of 1.16–7.63. In studies on American samples women scored higher than men, unlike studies on non-American samples where men scored higher than women (Lester, 2013).

The overall prevalence among medical students of anxiety disorders, the rate ranged from 29.2 to 38.7%, is higher than in the general population. The prevalence rate of eating disorders risk among medical students was found to be 10.4%, higher than in the general population, where it is about 5% (Treasure et al., 2010; Jahrami et al., 2019).

The most commonly used drugs by medical students are mainly alcohol (24%), tobacco (17.2%), and cannabis (11.8%), followed by hypnotic and sedative drugs (9.9%), stimulants (7.7%), cocaine (2.1%) and opiate (0.4%; Roncero et al., 2015). Male medical students presented a tendency to consume more of all types of drugs than females, with the exception of tranquilizers (Candido et al., 2018). Furthermore, it is reported that about half of the students experienced burnout during their undergraduate years (Ishak et al., 2013).

Among Italian medical students, according to Sampogna et al. (2020), the results showed a high prevalence of substance use, especially alcohol (range 13–86%), cigarettes (range 15–31%,), compared to Italian students from other degree programs. Cigarette use is also slightly higher than in the general population in Italy, which is around 20% (Lugo et al., 2017).

The prevalence rate of depression is around 20%, with depressive symptoms reported more frequently by female medical students. The prevalence of suicidal thoughts is around 17%, and is higher in men (Sampogna et al., 2020).

The evidence and importance of these data make it necessary to further research the possible bio-psycho-social factors that intervene in these age groups in determining the onset of psychological distress and psychic disorders. With the purpose of assessing determinants implicated in youth distress, we decided to focus on the attachment theory in our work, as it is increasingly considered and constantly evolving. Particularly, we dwelt on Parker’s construct of parental bonding, widely observed in clinical practice given its correlations with several disorders. Investigations into the relationship between childhood experiences and subsequent adult psychopathology suggest that negative parenting style create a diathesis for emotional and psychiatric dysfunction. Following a biopsychosocial approach to etiology and pathogenesis of psychopathology, parental bonding is one of the factors that might influence how a psychiatric disorder may develops. This diathesis, nevertheless, can be modified by a series of social and inter-personal experiences that have the capacity to neutralize the risk variable (Parker and Gladstone, 1996).

**Figure 1:**
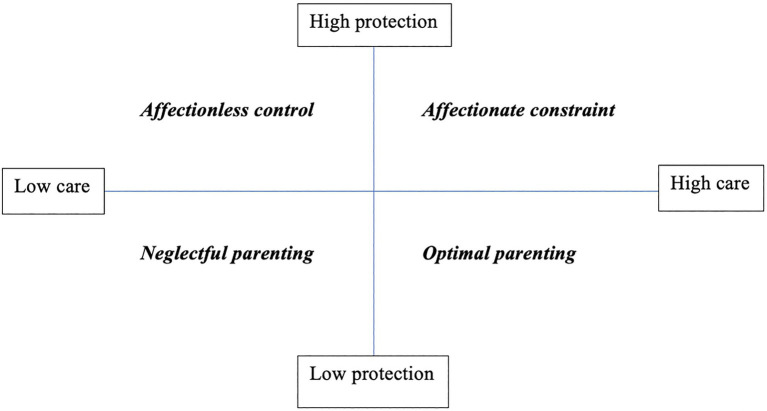
Parenting styles based on the combination of the dimensions “care” and “protection” (Parker et al., 1979).

Two main affective dimensions were highlighted among the characteristics of the parental educational style observed in practice: care and control (Roe and Siegelman, 1963; Schaefer, 1965; Raskin et al., 1971). The first of these two dimensions concerns the care and affection expressed by the parent, while the second one includes all the aspects of control, intrusiveness, and protection, understood as concern not related to affectionate feelings. Four different patterns of behavior and affective parenting styles are evidenced: low care/low protection (neglectful parenting), low care/high protection (affectionless control), high care/low protection (optimal parenting), and high care/high protection (affectionate constraint; Parker et al., 1979; Favaretto and Torresani, 1997; Figure 1).

The variety of studies investigating the correlation between PBI and psychiatric disorders agrees that of all the parental bonding styles, the one most implicated in psychopathology is affectionless control, characterized by poor parental care and high protection-control. This parental style is closely related to impaired formation of positive Internal Working Models (IWM), since due to low care and high parental control, the child struggles to develop a competent and worthy self-model and a reliable and supportive model of others (Otani et al., 2016). These compromised models will persist into adulthood, making the individual more susceptible to the development of psychiatric disorders (Parker and Gladstone, 1996).

Using the PBI, different studies indicated affectionless control as related to major depression (Parker, 1979; Enns et al., 2002; Heider et al., 2006; Visioli et al., 2012), anxiety disorders (Silove et al., 1991; Heider et al., 2008), alcohol and drug use disorders (Kendler et al., 2000; Torresani et al., 2000) and personality disorders (Reti et al., 2002; Russ et al., 2003). Other conditions related to a parental style characterized by low care and high control are eating disorders and obesity (Turner et al., 2005; Amianto et al., 2021), obsessive compulsive disorders (Myhr et al., 2004; Wilcox et al., 2008; Lennertz et al., 2010; Chen et al., 2017), sleep disorders (Shibata et al., 2016), chronic pain (Anno et al., 2015) but also schizophrenia (where it is also associated with a poor prognosis; Parker, 1982; Parker et al., 1988; Winther Helgeland and Torgersen, 1997; Favaretto et al., 2001; Willinger et al., 2002) and individuals at ultra-high risk (UHR) for psychosis (Peh et al., 2020).

Several studies highlighted the relationship between poor parental bonding and suicidal ideation and suicidal behavior (Miller et al., 1992; Adam et al., 1994; Martin and Waite, 1994; McGarvey et al., 1999; Yamaguchi et al., 2000; Lai and McBride-Chang, 2001; Diamond et al., 2005; Dale et al., 2010; Freudenstein et al., 2011). Recently, Siqueira-Campos et al. (2021), in a sample of medical students, showed that there are significant independent associations between maternal affectionless control and depression, between maternal negligent parenting and depression, and between paternal affective constraint and suicidal ideation. This study suggests also that depression influences the association between maternal affectionless control and anxiety and the association between maternal affectionless control and suicidal ideation.

Regarding maternal bonding, low care and more often affectionless control have been found to be significantly associated with higher levels of suicidality, leading to the conclusion that this type of parental bonding could be considered a specific (direct or indirect, it is not yet clarified) risk factor for suicidality. Concerning paternal bonding, the data are less consistent, suggesting that also here low care and often affectionless control are associated with an increased risk of suicide (Goschin et al., 2013). Discrepancies between mother and father may be related to cultural differences in the father’s role in the upbringing and control of the offspring. We can therefore conclude that affectionless control of the parent who is more invested in the child’s growth is correlated with an increased suicide risk.

Given this background, we chose to evaluate the relationship between the quality of perceived parenting style with psychological well-being of medical students in Italy since there was no Italian study with the same characteristics in the literature.

We decided to give gender a certain relevance in our study since it is often not taken into account in the literature regarding attachment, and furthermore the data in the field are few and discordant, probably due to limitations and design of the studies. Adam et al. (1994) found that in suicidal female adolescents both father and mother are perceived as affectionless and overcontrolling, whereas in males only the mother. Kovess-Masfety et al. (2011), however, reported that males with suicidal ideation and attempts perceived their mothers as affectionless controlling, while fathers only as low caring, while McGarvey et al. (1999) reported that among suicidal males paternal affectionless control was reported more than maternal affectionless control. Other studies have shown that in suicidal females the perception of an affectionless control style is related to both parents (Goldney, 1985; Yamaguchi et al., 2000) or only to the mother (Diamond et al., 2005).

Under these premises, we set up a cross-sectional study with the aim to explore the psychological condition of medical students, with a specific emphasis on depression and suicidal ideation, in order to identify correlations between these two clinical variables and the quality of parental bonds. Gender differences were also considered in the descriptive and correlational analysis since literature data on the topic were few and discordant. Therefore, we hypothesized that our sample of medical students would have higher rates of suicidal ideation and depression than the general population, especially the students who had a parental bonding characterized by low care and high control (affectionless control) or low care and low control (neglectful parenting). Moreover, we assume that gender differences relative to the impact of parental bonding on suicidal ideation and depression could be find.

## Materials and methods

### Participants

The sample of this study includes students (first to sixth year, including out-of-course students) of the Faculty of Medicine of the University of Ferrara, Italy. The study was publicized through the use of electronic media (university email and student message group) and the data collection was based on a questionnaire created in Google Forms. The choice of the Google Forms platform was based on the simplicity of its use (and thus reduce to zero any errors in compilation), and because we expected that an online data collection would guarantee a higher number of participants, creating in this way an adequate sample size. Participation in the research was guaranteed by anonymity: data collection on Google Docs generated a database with numerically encoded information, without any possibility of tracing the identity of the respondent. Recruitment of respondents began on July 20, 2021, and ended on September 12, 2021, in order to ensure more students responded to the questionnaire given the exam period and summer vacation. To ensure that only students enrolled in the medical school would fill it out, we made sure that only those who had received the email from the educational coordinator had access to the questionnaire. Students could only access the questionnaire through exclusive use of their credentials accepted by the University of Ferrara’s IT management system. In addition, no underage subjects were included among the participants, as compulsory schooling in Italy lasts one year longer than in other countries, and consequently students access university when they reach the age of majority.

The study received approval from the Ethics Committee of University of Ferrara (Italy).

### Procedure

The study was conducted by the Neurological, Psychiatric, and Psychological Sciences Section of the Department of Neuroscience and Rehabilitation, Faculty of Medicine, Pharmacy and Prevention, University of Ferrara (Italy). Demographic and anamnestic data and psychometric measures were retrieved from an online questionnaire, which was delivered through the Google Forms platform.

Before beginning the questionnaire, there was a brief description of the study and its instruments to inform and guide the respondent through the completion.

### Measures

#### Personal data sheet

For each participant, the following information was collected: age, sex, year of course, age of father and mother, presence or absence of siblings in the family and some anamnestic data on psychological health: whether they had experienced psychological distress with impairments in quality of life, poor performance in daily activities or obstacles in life choices; whether they had used psychopharmaceutical drugs or had undergone psychotherapy sessions; whether they had ever used drugs or alcohol, and whether they were currently abusing drugs or alcohol. It was also assessed if any participants had experienced self-injurious behaviors or suicide attempts.

The last two questions in the personal data sheet were about recent life events that have negatively affected psychological condition and quality of life, as follows: “In the last six months, has an event occurred that has negatively affected your psychological condition and quality of life?” and “If YES, which one or ones?,” allowing respondents to choose from the following answers: “The death of a loved one,” “a failure in my academic career,” “a problem in my family of origin,” “an economic problem,” “a sentimental problem, the end of an emotional relationship,” “the separation from my family and/or my country” and “other.”

#### Parental bonding instrument

The PBI is a self-administered questionnaire that consists of two forms, one for the father and one for the mother, each with 25 items: 12 items assess the “care” dimension, which implies an affectionate and empathetic attitude, while the remaining 13 assess the “protection” dimension, which implies controlling and constraining behaviors. Based on how children remember their parents in their first 16 years of life, they will assign a rating to the different statements contained in the items. The score is assigned according to a Likert scale with values from 0 to 3 for each statement, so the total score will have a range 0–36 for the “care” dimension and 0–39 for the “protection” dimension. For the “care” dimension, if the score is 24 or higher (for the father) or 27 or higher (for the mother) it will be called “high care,” if lower “low care.” For the dimension “protection,” a score equal to or greater than 12.5 (for the father) and 13.5 (for the mother) indicates “high protection,” if lower “low protection.” There are four possible patterns of behavior and affective parenting style depending on the combinations of the two dimensions: low care/low protection (neglectful parenting), low care/high protection (affectionless control), high care/low protection (optimal parenting) and high care/high protection (affectionate constraint). The PBI is an instrument that has shown excellent test–retest reliability and durability over time, even at 10 and 20 years (Parker et al., 1979; Parker, 1982; Warner and Atkinson, 1988; Mackinnon et al., 1989; Wilhelm et al., 2005; Murphy et al., 2010).

In the current study it was used the Italian version of the PBI, which reports in the sample of university students the following mean values of “care” and “protection”: for the mother the “care” mean score was 29.81 (±6.15) and for the father 26.80 (±7.87); regarding “protection,” the mean scores were 13.79 (±7.38) for the mother and 12.41 (±6.96) for the father. In male students, the mean scores were for “mother care” 31.21 (±4.59) and “father care” 27.9 (±7.56); while for “mother protection” 12.17 (±6.01) and “father protection” 11.89 (±5.65). In female students, the mean scores were for “mother care” 27.65 (±7.55) and “father care” 25.1 (±8.13); while for “mother protection” 16.3 (±8.6) and “father protection” 13.55 (±8.61; Scinto et al., 1999). In the sample of students of the validation study, Cronbach’s alpha was 0.88 and 0.86 (respectively, care and protection) for the mother, while 0.91 and 0.83 (respectively, care and protection) for the father. In our sample, Cronbach’s alpha was 0.92 and 0.88 (respectively, care and protection) for the mother, while 0.92 and 0.87 (respectively, care and protection) for the father.

#### Beck hopelessness scale

The BHS is a 20-item self-rating scale that detects and quantify “hopelessness,” that is a negative attributional attitude about future possibilities, included in Beck’s cognitive model of depression (Beck, 1967), associated with increased suicidal risk and related to negative feelings about the future, loss of motivation, and loss of expectations (Beck et al., 1974). This scale assesses the severity of negative expectations about the future in both the short and long term. It evaluates the respondent’s feelings over the previous week using “True/False” responses corresponding to a score of 0 or 1. The total score ranges from 0 to 20 and higher scores indicate a higher prevalence of suicidal ideation. Of the 20 true-false statements, 9 are FALSE bound and 11 are TRUE bound to indicate the presence of pessimism in the future. The BHS takes 5–10 min to complete, and where required can also be administered orally by the examiner. It is recommended for individuals over the age of 17 (Pompili et al., 2009).

This instrument has demonstrated particular utility as an indirect indicator of suicide risk in depressed individuals or individuals who have attempted suicide, and although it was not developed as an instrument to determine hopelessness in adolescents and adults in the general population, it has nevertheless been used for these purposes as well (Greene, 1981; Durham, 1982).

In this study we used the Italian version of the BHS (Pompili et al., 2009). The internal consistency reliability of the BHS measured using the KR-20 index (Kuder–Richardson Formula, analogous to Cronbach’s alpha for dichotomous measures) ranges between 0.87 and 0.93 for the original version (Beck and Steer, 1993). In the Italian version, the KR-20 index ranges between 0.75 (university student sample) and 0.89 (psychiatric sample; Pompili et al., 2009). In our sample, Cronbach’s alpha was 0.73.

#### Beck depression inventory – II

The BDI-II is the most widely used instrument for detecting the existence and severity of depressive symptoms taking into account affective, cognitive, somatic, and vegetative domains (Beck et al., 1996). It is based on Beck’s theory that depressed patients are characterized by a negative triad, i.e., negative representations of themselves, of the present and of the future (Beck, 1967).

It is a self-administered tool containing 21 items, each using a 4-point scale, that takes around 5–10 min to complete. It can be used with individuals aged 13 and above (Beck et al., 1996). The patient is asked to consider each statement relating to the way he or she has felt over the past 2 weeks. The following domains are evaluated: sadness, pessimism, past failure, loss of pleasure, guilty feelings, punishment feelings, self-dislike, self-criticalness, suicidal thoughts or wishes, crying, agitation, loss of interest, indecisiveness, worthlessness, loss of energy, changes in sleeping pattern, irritability, changes in appetite, concentration difficulty, tiredness or fatigue, and loss of interest in sex. Scores in each item range from 0 (absence of symptoms) to 3 (severe symptoms) and the total score ranges from 0 to 63. Higher scores indicate more severe depressive symptoms.

Through the questionnaire we are able to have information related to individual items, which can be of help to the clinician. One of them that we decided to take into consideration is item #9 “Suicidal thoughts,” which has been shown to be indicative of suicidal ideation and suicide risk (Green et al., 2015).

In our study we used the Italian version of the BDI-II (Ghisi et al., 2006), using a cut-off score ≥ 14 as the threshold for detecting a clinically significant presence of depressive symptoms, with the following score ranges to quantify the severity of the depression: 0–13 minimal, 14–19 mild, 20–28 moderate, and 29–63 severe (Beck et al., 1996). The BDI has proven to be an excellent case-finding screen for depression in a variety of adult samples. However, in the general population the cut-scores should be adapted to the sample because those previously listed refer to a population of patients with a diagnosis of major depression, and therefore are designed to have few false negatives (Hubley, 2014). Furthermore it should be noted that when interpreting the results we should always keep in mind that we are using a screening instrument, and therefore the diagnosis of depression requires further analysis and that we may have response bias (with over- and under-reported symptoms; Hubley, 2014).

Cronbach’s alpha for the BDI-II is 0.87 and the split-half reliability coefficient is 0.77. In our sample, Cronbach’s alpha was 0.92.

### Data analysis

Data were presented as absolute numbers, percentages, mean ± Standard Deviation (SD) if normally distributed, or median and interquartile ranges (IQR) as appropriate on the basis of data distribution. Comparisons were performed using a two-tailed, independent samples student t-test or Mann Whitney U test as appropriate, according to the data distribution for continuous variables. Dichotomous variables were compared using the Chi squared test. The correlation between variables was tested by calculating the Spearman’s correlation coefficient. To compare BDS and BHS scores across different categories of parental bonding, we used the Kruskal–Wallis test. To identify variables independently associated with the probability of scoring positive either on BDS or BHS, we calculated the odds ratio (OR) and 95% confidence interval (CI) by means of multivariable logistic regression analysis. In two logistic regression models, dichotomous BDS and BHS were entered as dependent variable. The two models included: sex, age, year of course, “Mother Care,” “Father Care,” “Mother Protection,” and “Father Protection.” Age and year of course were entered as continuous variables, the others as dichotomous variables. Moreover, the same analyses were carried out separately for males and females. The program used for the analysis was SPSS version 25.

## Results

### Descriptive analysis

**Figure 2:**
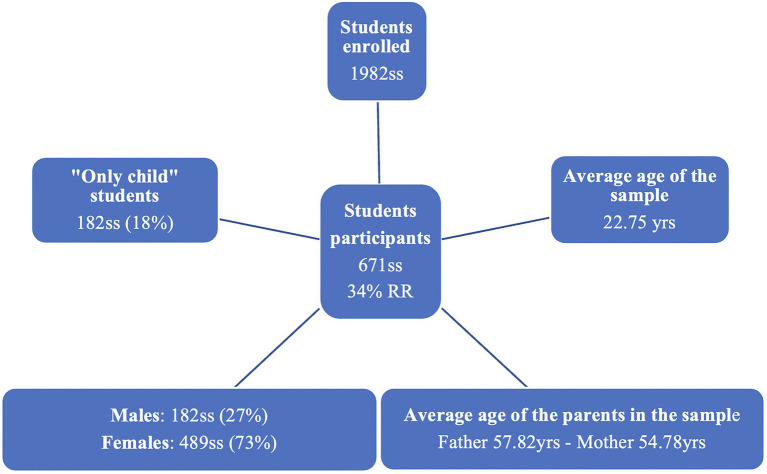
Sample data.

The total number of students registered in the medical school at the university was 1982, while the number of students who responded to the questionnaire was 671. The response rate (RR) was therefore 34%. Of these 671, 489 were females (72.9%) and 182 were males (27.1%). The average age of the sample was 22.75 (±3.525). Regarding the year of the course, the students were divided as follows in the sample: 176 of the first year (26.2%), 199 of the second (29.7%), 66 of the third (9.8%), 71 of the fourth (10.6%), 69 of the fifth (10.3%), 35 of the sixth (5.2%) and 55 out-of-class students (8.2%). Average age of the father in the sample was 57.82 (±6.02) and of the mother 54.78 (±5.3). To the question “Are you an only child?” 548 participants answered “No” (81.7%) and 122 with “Yes” (18.2%; Figure 2).

**Figure 3:**
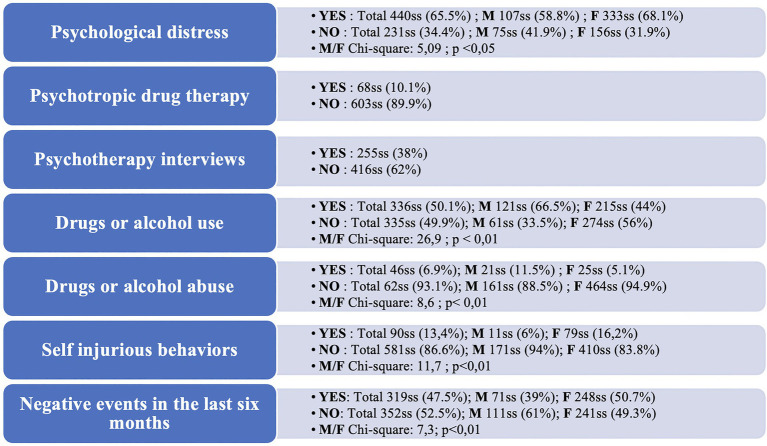
Anamnestic data.

About the descriptive analysis of the anamnestic portion of the first battery of questions, the results are as follows (Figure 3).

Within our sample, to the question “Have you ever experienced psychological distress such that you felt your quality of life was significantly altered, encountered obstacles in your life choices, and poor performance in your activities?” 440 participants (65.6%) answered “Yes” while 231 “No” (34.4%). Among the “Yes” respondents, more women (68.1% among females) than men (58.8% among males) reported this distress, and the difference was statistically significant (Chi square = 5.09; *p* < 0.05).

When asked “Have you ever had psychotropic drug therapy in the past?” 603 participants (89.9%) said “No,” whereas 68 (10.1%) answered “Yes.” We found no significant gender differences in this item.

To the question “Have you had psychotherapy interviews in the past?” 416 participants (62%) said “No,” while 255 or 38%, answered “Yes.” Again, we found no significant gender differences.

Regarding substance use and abuse, to the question “Have you ever used drugs or alcohol?” 49.9% of respondents (335) answered “No,” while 50.1%, or 336 respondents, answered “Yes.” In this case, as evidenced by the contingency table, we found a statistically significant gender difference (Chi square 26.9; *p* < 0.01), with 66% of male respondents versus 44% of female respondents reporting drug or alcohol use.

To the question “Do you currently tend to use drugs or alcohol with consequences for your performance ability?” 6.9% of the sample (46 individuals) responded positively, while 93.1% (625 individuals) responded negatively. Also here, as in the previous question, and as evidenced by the contingency table, we found a statistically significant gender difference (Chi square 8.6; *p* < 0.01), with 11.5% of males answering affirmatively to the item, compared to 5.1% of females.

When asked “Have you ever experienced self-injurious behaviors supported by suicidal ideation?” 581 respondents (86.6%) answered “No,” while 90 (13.4%) answered “Yes.” As evidenced by the contingency table, we found a statistically significant gender difference in responses to this item (Chi square 11.7; *p* < 0.01), with 16.2% of female respondents responding positively, compared with 6% of males.

Regarding the last two items, to the question “In the last six months did an event occur that negatively affected your psychological condition and quality of life?,” 52.5% of respondents (352 individuals) answered “No,” while 47.5% (319ss) answered “Yes.” We found a statistically significant gender difference in this item as well (Chi square 7.3; *p* < 0.01), with more females (50.7% of females) than males (39% of males) reporting a recent negative event. Among those who answered “Yes” to the last item (“In the last six months did an event occur that negatively affected your psychological condition and quality of life?”), it was also asked to select which of the events on the list had negatively impacted quality of life and psychological status. We left the option of selecting more than one response for this item. These were the percentages of the selected items: “Other” (42%; 136ss), followed by “A failure in my academic career” (33.3%; 108ss), “A problem with my family of origin” (22.5%; 73ss), “A sentimental problem, the end of an affective story” (21%; 68ss), “The death of a beloved person” (18,2%; 59ss), “An economic problem” (10.2%; 33ss) and finally “The separation of my family and/or my country”(4%; 13ss).

About the PBI, the results are as follows.

Referring to the father, the average score on the dimension “care” was 22.38 (±8.42), while on the dimension “protection” it was 11.94 (±7.14), whereas considering the mother, the mean score related to the dimension “care” was 27.47 (±7.4), while for the dimension “protection” it was 14.09 (±7.70).

Referring to the father, on the “care” dimension 48.7% (327ss) of respondents showed “Low Care,” while 51.3% (344ss) “High Care”; on the “protection” dimension, 60.8% (408ss) of respondents showed “Low protection,” while 39.2% (263ss) “High protection.” Considering the mother, on the “care” dimension 33.8% (227ss) of respondents showed “Low Care,” while 66.2% (444ss) “High Care”; on the “protection” dimension, 51.7% (347ss) of respondents reported “Low protection,” while 48.3% (324ss) “High protection” (Table 1).

**Table 1:** Percentages concerning high or low “care” and “protection” of the parents in the sample.

		Father		Mother	
		Frequency	Percentage	Frequency	Percentage
Care	High	344	51.30%	444	66.20%
	Low	327	48.70%	227	38.80%
Protection	High	263	39.20%	324	48.30%
	Low	408	60.80%	347	51.70%

Regarding the categorization of father’s parenting, 14.9% of the sample showed “Affectionate constraint,” 36.4% “Optimal parenting,” 24.3% “Affectionless control” and 24.4% “Neglectful parenting,” whereas the mother’s 23.4% of the sample reported “Affectionate constraint,” 42.8% “Optimal parenting,” 24.9% “Affectionless control” and 8.9% “Neglectful parenting.” For both high and low protection, as well as parenting categories, in relation to both parents, we found no significant gender differences (Table 2).

**Table 2:** Percentages related to parenting style groups in the sample.

	Father		Mother	
	Frequency	Percentage	Frequency	Percentage
Affectionate constraint	100	14.90%	157	23.40%
Optimal parenting	244	36.40%	287	42.80%
Affectionless control	163	24.30%	167	24.90%
Neglectful parenting	164	24.40%	60	8.90%

In the BHS, and referring to the score, the median was 4 with an Interquartile Range (IQR) 2–7. No statistically significant gender differences were evident in this case. Concerning the cut-off, participants who had a score greater than or equal to 9 were 112 (16.7%), while 8 or less were 559 (83.3%). Again, no statistically significant gender differences were found regarding the cut-off.

In the BDI-II, referring to the score, the median was 14, with an interquartile range 7–22 (Table 3). In this case, however, we found a statistically significant difference (*p* < 0.01) in gender, as the median of females (14; IQR 9–23) was higher than the median of males (9; IQR 5–17.25).

**Table 3:** BHS and BDI-II score/cut-off statistics.

	BHS			BDI-II		
Score	Median	4			14	
	IQR	2–7			7–22	
Cut-off	≥9	Frequency	112	≥14	Frequency	337
		Percentage	16.70%		Percentage	50.20%
	≤8	Frequency	559	≤13	Frequency	334
		Percentage	83.30%		Percentage	49.80%

When we refer to the cut-off, 50.2% of the sample (337ss) showed a possible depressive disorder because they scored 14 or higher, while 49.8%, (334 respondents) scored 13 or lower (Table 3). We again showed a statistically significant gender difference (*p* < 0.01): 54.19% of females exceeded the cut-off, in contrast to 39.56% of males.

On question #9 of the BDI-II, “Suicidal thoughts,” 17.73% of our sample (119 responses) scored 1 or higher, indicating suicidal ideation, while 82.27% (552) had a score of 0. We found no statistically significant gender differences.

Because BHS also assesses suicidal ideation, we wanted to compare this item with median scores, and the #9 BDI-II cut-off. Among respondents who scored 1 or higher on item #9 of the BDI-II the median BHS score was 8 (IQR 4–13), whereas among those who scored 0 the median BHS score was 3(IQR 1–6). This difference was statistically significant (*p* < 0.01).

### Correlation analysis

The BHS score, in the general sample, has several statistically significant correlations (*p* < 0.01): it correlates negatively with the “Mother Care” (Spearman’s Rho = −0.275), “Father Care” (Spearman’s Rho = −0.196) score and positively with the “Mother Protection” (Spearman’s Rho = 0.221), “Father Protection” (Spearman’s Rho = 0.118) and “BDI-II” (Spearman’s Rho = 0.600) score. These correlations remained statistically significant even when analyzing between the two genders (Table 4).

**Table 4:** Nonparametric correlations between father’s and mother’s “care” and “protection” and BHS and BDI-II scores.

			Mother’s care	Mother’s protection	Father’s care	Father’s protection	BHS score	BDI-II score
Spearman’s rho	BHS score	Correlation coefficient	−0.275	0.221	−0.196	0.118	1.000	0.600
		Sig. (*p*)	0.000	0.000	0.000	0.002	–	0.000
	BDI-II score	Correlation coefficient	−0.341	0.267	−0.209	0.125	0.600	1.000
		Sig. (*p*)	0.000	0.000	0.000	0.001	0.000	–

The BDI-II score, in the overall sample, has several statistically significant correlations (*p* < 0.01): it correlates negatively with the “Mother Care” (Spearman’s Rho = −0.341), “Father Care” (Spearman’s Rho = −0.209) score and positively with the “Mother Protection” (Spearman’s Rho = 0.267), “Father Protection” (Spearman’s Rho = 0.125) and “BHS” (Spearman’s Rho = 0.600) scores (Table 4). These correlations remained statistically significant even in the between-gender analysis.

BHS and BDI scores significantly differed in the four categories of PBI.

For Parenting Father categories, we found a significant difference for both BHS (*p* < 0.001) and BDI-II (*p* < 0.001) scores. At the *post hoc* analysis, regarding BHS, the significant differences were between the category “Optimal parenting” and “Affectionless control” (*p* < 0.001) and between “Optimal parenting” and “Neglectful parenting” (*p* < 0.005). In fact, for the “optimal parenting” group the median BHS score was 3 (IQR 1–5) while for the “Affectionless control” category the median was 4 (IQR 2–8) and for “Neglectful parenting” it was 4 (IQR 2–7; Table 5).

**Table 5:** BHS/BDI-II scores divided among parenting groups.

		Father		Mother	
		BHS	BDI-II	BHS	BDI-II
Affectionate constraint	Mean	4.50	13.26	4.56	14.20
	Median	3.00	12.00	4.00	13.00
	SD	4.123	8.724	3.921	9.068
	IQR	1–7	7–16	2–6	7.50–19
Optimal parenting	Mean	3.91	13.52	3.40	11.86
	Median	3.00	11.50	3.00	10.00
	SD	3.808	10.205	3.209	9.424
	IQR	1–5	6–19	1–5	5–16
Affectionless control	Mean	5.74	18.79	6.51	21.68
	Median	4.00	16.00	5.00	20.00
	SD	5.172	12.304	5.095	11.887
	IQR	2–8	9–27	3–9	13–30
Neglectful parenting	Mean	5.12	16.70	6.65	19.53
	Median	4.00	14.50	5.00	18.00
	SD	4.150	11.357	5.514	12.168
	IQR	2–7	8–23	2–10	9.25–25.75

Still in the *post hoc* analysis, regarding BDI-II, the significant differences were between “Affectionate constraint” and “Affectionless control” (*p* < 0.05), “Optimal parenting” and “Affectionless control” (*p* < 0.005) and between “Optimal parenting” and “Neglectful parenting” (*p* < 0.05). In fact, for the “Affectionate constraint” group, the median BDI-II score was 12 (IQR 7–16), for “Optimal parenting” 11,50 (IQR 6–19), for “Affectionless control” 16 (IQR 9–27) and for “Neglectful parenting” 14,50 (IQR 8–23) (Table 5).

By analyzing separately the two genders, we found the above significant differences only for women (*p* < 0.001 for both BHS and BDI). There were no statistically significant differences regarding male sex.

Regarding Parenting Mother categories, we again found a significant difference for both BHS (*p* < 0.0001) and BDI-II (*p* < 0.0001) scores.

At *post hoc* analysis, regarding BHS, the significant differences were between “Affectionate constraint” and “Optimal parenting” (*p* < 0.05), “Affectionate constraint” and “Affectionless control” (*p* < 0.0001), “Optimal parenting” and “Affectionless control” (*p* < 0.001) and between “Optimal parenting” and “Neglectful parenting” (*p* < 0.05). In fact, for the “Affectionate constraint” group the median BHS score was 4 (IQR 2–6), “Optimal parenting” 3 (IQR 1–5), “Affectionless control” 5 (IQR 3–9) and “Neglectful parenting” 5 (IQR 2–10; Table 5).

Relative to BDI-II, *post hoc* analysis showed that the significant differences were between “Affectionate constraint” and “Optimal parenting” (*p* < 0.05), “Affectionate constraint” and “Affectionless control” (*p* < 0.0001), “Affectionate constraint” and “Neglectful parenting” (*p* < 0.05), “Optimal parenting” and “Affectionless control” (*p* < 0.0001), and between “Optimal parenting” and “Neglectful parenting” (*p* < 0.0001), In fact, for the “Affectionate constraint” group the median BDI-II score was 13 (IQR 7,50–19), for “Optimal parenting” 10 (IQR 5–16), for “Affectionless control” 20 (IQR 13–30) and for “Neglectful parenting” 18 (IQR 9,25-25,75; Table 5).

The differences remained statistically significant analyzing men (*p* < 0.05 for BHS, and *p* < 0.0001 for BDI) and women (*p* < 0.0001 for both scores).

Moreover, “Father Care” (OR = 0.97; C.I. 95% 0.94–0.99), and “Mother Care” (OR = 0.95; C.I. 95% 0.92–0.98) predicted a lower probability to screen positive at the BHS, in the total sample and in women. As for BDI-II, female gender was significantly associated with the probability to have a “positive” BDI (OR 1.84; C.I. 95% 1.27–2.67), as did the “Mother Protection” score (OR 1.05; C.I. 95% 1.02–1.08). “Father Care” (OR 0.96; C.I. 95% 0.94–0.98) and “Mother Care” (OR 0.96; C.I. 95% 0.93–0.98) are “protective factors.” By running separately, the same analysis by sex, only “Mother Protection” retained statistical significance (OR 1.09; C.I. 95% 1.03–1.15) in men. In women, we confirmed the same association as in the total sample: “Father Care” (OR 0.96; C.I. 95% 0.93–0.98), “Mother Care” (OR 0.96; C.I. 95% 0.93–0.98) and “Mother Protection” (OR 1.04; C.I. 95% 1.01–1.07).

## Discussion

Through this discussion, by taking up and summarizing the results and comparing them with the existing literature on these topics, we will try to dwell both on the confirmations we have received as they add statistical relevance to the concepts, and on the novelty aspects that characterized the work.

To begin with, the results obtained from the personal data sheet showed us significant gender differences. More females than males suffer a distress affecting their quality of life, with self-injurious behaviors sustained by suicidal ideas and reported a negative event affecting their psychological condition. On the other hand, more males than females reported use or abuse of drugs or alcohol. This last data is in line with the current literature, which shows a higher percentage of males than females in alcohol and drugs use/abuse among medical students (Candido et al., 2018).

Another interesting finding was “a failure in my academic career” as the main specific negative event impacted over psychological status and quality of life of participants. The finding is consistent and understandable given the specificity of the sample studied but should not be underestimated.

Results in our sample using BHS to assess suicidal ideation are consistent with the finding in Lester’s 2013) review of hopelessness in college students. Nonetheless, the percentage of participants who exceeded the cut-off value indicative of suicidal ideation (16.7%) was higher than that reported in the Rotenstein et al.’s (2016) meta-analysis regarding the prevalence of suicidal ideation among medical students (11.1%) and also exceed the overall lifetime prevalence of suicidal ideation, which is 9.2% (Nock et al., 2008). Assessment with item #9 of the BDI-II likewise found the presence of suicidal ideation in nearly one in five students. However, our data concurs with that of Sampogna et al. (2020) about the percentage of suicidal thoughts in Italian medical students, which was reported to be around 17%. Because our finding agrees with the Italian data but differs from those abroad and in the general population, the evidence adds statistical significance to the psychological condition of medical students in Italy.

Regarding depressive symptoms in medical students our study reports high prevalence rates, with more than a half of the sample who exceeded the BDI-II cut-off. This data differs from Rotenstein et al. (2016), which reported that the median summary prevalence among medical students was 32.4% (95% CI, 25.8–39.7%) for the Beck Depression Inventory (BDI) with a cut-off score of 10 or greater (the cut-off of 10 in the BDI is comparable to that of 14 in the BDI-II). As seen earlier regarding suicidality, again the Italian finding is higher than the foreign one.

Our findings also confirm the well-known evidence of gender differences in reporting the presence of depressive symptoms, with female gender at higher risk of developing depression (Malhi and Mann, 2018).

Our sample therefore showed higher prevalence rates of suicidal ideation and depression than the data in the literature and the general population. This finding is also relevant as it could be related to the mental health consequences of the SARS-CoV2 pandemic, even though our data collection occurred in the summer, a season with lower percentages of Covid19 cases and less psychological distress due to restrictions.

About parenting styles our study showed mean scores in the two dimensions of “Care” and “Protection” quite in line with those found on a sample of Italian university students in Scinto’s study (Scinto et al., 1999) with the evidence of a trend already highlighted by the literature that mothers are rated as more caring and protective than fathers (Parker et al., 1979; Parker, 1983; Truant et al., 1987).

With respect to the impact that the quality of parental bonds has on suicidal ideation and depression, we received several confirmations, plus some new evidence.

First, in BHS data analysis, score correlates positively with BDI-II score and with the “protection” dimension, and negatively with the “care” dimension. Similarly, we found that the BDI-II score correlates positively with the BHS score and with the “protection” dimension, and negatively with the “care” dimension. These correlations agree with the current literature where we saw that affectionless control, the pattern of parenting characterized by low care and high control, is related to suicidal ideation (Goschin et al., 2013) and major depression (Parker, 1979; Enns et al., 2002; Heider et al., 2006; Visioli et al., 2012). Furthermore, the correlation between BDI-II and BHS confirms how depressive symptoms play a crucial role in suicidal ideation (Klonsky et al., 2016).

Secondly, considering the associations between BHS and BDI-II scores with the four Parental Bonding styles evidenced by the PBI, we found significant differences. Regarding the father, “Affectionless control” and “Neglectful parenting” related to higher BHS and BDI-II scores than “Optimal parenting.” Once the same analysis was done individually on the two genders, these differences were no longer relevant, but remained only among females. About the mother, with respect to BHS and BDI-II, we found more statistically significant differences between the four patterns than in the father.

As for BHS, in addition to “Affectionless control” and “Neglectful parenting,” we also detected a higher average score of BHS in “Affectionate constraint,” perhaps indicating the importance of the role of the “Protection” dimension suicidal ideation’s onset. Likewise the average scores for BDI-II were higher in “Affectionless control” and “Neglectful parenting” than in “Affectionate constraint” and “Optimal parenting.”

If we consider the gender comparison, findings highlighted significant differences in males and females. Il appeared to us that males were less sensitive to father parenting with regard to suicidal ideation and depression. Furthermore, the mother’s parenting, in both males and females, seemed to have a greater impact than the father’s in relation to the BHS and BDI-II scores. This remark agrees with those made in the review of Goschin et al. (2013).

Thirdly, we found that the “Care” dimension of father and mother was a “protective factor” toward suicidal ideation in the overall sample, but once the analysis is differentiated for gender, this dimension remains “protective” only in females. As for depressive symptoms, female gender was the most relevant predictor, along with “Mother protection” as well. Again, the “care” dimension of father and mother proved to be a “protective factor” for depressive symptoms. In the two-gender analysis, however, the dimension “care” of father and mother is not more “protective” in males, but only in females.

This discordance between males and females on how the quality of their parental bond affects suicidal ideation and depression, suggests that perhaps males are less sensitive than females to Parental Bonding. This point in particular is a novelty in our work, which could be a starting point for future studies on several grounds: on one hand, to find confirmation of this statement, and on the other, to investigate its reasons and evolution. The significance of the finding is possibly related to sociocultural factors and the predominant role that the mother has played and still plays in child growth in Italian context, although there have been changes in the last years. Imagining a study similar to ours in the next years might in fact show changes that would help us understand and analyze more carefully the factors involved in psychopathology.

This study has some limitations. In the sample, males are substantially less represented than females (27.1% vs. 72.9%). In addition, cross-sectional studies are not adequate to test etiological hypotheses but only to formulate them and are susceptible to biases such as responder bias, recall bias, interviewer bias and social acceptability bias. The use of self-rated psychometric instruments may be susceptible to cognitive bias, bias of overestimation or underestimation of symptoms, or in the case of PBI, since retrospective, even to memory bias. Finally, another limitation of this study is the absence of a control group.

## Conclusion

These data are partial and preliminary, so more studies are necessary in order to further expand knowledge on this topic. Nevertheless, we may consider some clinical implications of the work, and suggest some recommendations to students, families, universities, and society.

From a clinical perspective, the work suggests that we should pay attention, in assessing depressive symptomatology and suicidal ideation, to the patient’s relationship with his or her family. Moreover, in focusing on this determinant, it is good to take into account the gender differences that might be observed.

Furthermore, even in our study we emphasized the higher impact of mother’s bad parenting on the susceptibility and onset of depression and suicidal ideation. Nevertheless, it is good to take into consideration the figure of the father, who might over the years get a stronger impact on the psychopathology as a result of sociocultural changes concerning the roles and functions of the father figure in the family setting and in the specificity of the Italian context. This is precisely why the PBI could help, as an agile tool that allows us in a relatively short time to have important insights into both parenting styles.

The recommendations to be made are therefore many. As for students, the decision to undertake a long and difficult course of study such as Medicine should be more individual and independent and less related to family or cultural heritage. Moreover, it is interesting to point out that the choice to pursue this type of career is probably linked to original narcissistic wounds, and consequently to reparative drives (“caring for others to care for oneself”) that may manifest themselves in the choice of a “helping profession,” particularly the medical one. It would be a good idea, therefore, to activate counselling services starting from high schools, so as to wisely guide students to a more conscious choice for their future.

With regard to parents, however, the importance of care and of limiting control is significant and should also be combined with better harmony and cooperation between parental figures.

Universities should try to strengthen specific counselling services, and to conduct student evaluations that are as integrated and comprehensive as possible. Given the onset of major psychiatric illnesses precisely in this stage of life, it is necessary to intercept the specific vulnerabilities of this juvenile population early, to capture the individual reasons underlying psychological distress, and to diagnose the existence of psychiatric disorders. Strengthening these interventions would allow us to intervene preventively, limiting the recurrence and the amplitude of negative outcomes. So, it might be worthwhile in the first two years of the degree to submit all students to psychiatric screening evaluations by means of quickly and easily administered psychometric self-assessment instruments, which, although limited in their reliability, can provide interesting and useful information in order to prevent the onset of psychological distress.

Concerning the society, the study confirms that in Italy the percentages of depressive symptoms and suicidal ideation in medical students are higher than in other countries. This could stimulate a reflection on what the differences are in the conditions of our students compared to those abroad, trying to draw lessons from other realities, which could increase students’ wellbeing.

Our study also found that, among students, a high percentage (33%, 1 in 3 students) reported an academic failure as an event that worsened their quality of life and negatively impacted their psychological health. The sometimes more social than personal need and drive to “succeed” could be another key to interpret these results.

University students, today more than in the past, have a specific vulnerability to the psychological stress due to performative demands they are subjected to. This, along with the reality that the onset of many psychiatric disorders occurs precisely between adolescence and early youth, prompts us to dwell on the importance for universities, families, and society to focus on the quality of life and mental health of their students. While it is important to maintain a meritocratic mechanism of “rewarding” work through grades, at the same time, the necessity of supporting students in their academic journey with proper attention to their mental condition must be emphasized.

## Data availability statement

The raw data supporting the conclusions of this article will be made available by the authors, without undue reservation.

## Ethics statement

All procedures performed in the study were in accordance with the 1964 Helsinki declaration and its later amendments or comparable ethical standards, and were approved by the Ethics Committee of the University of Ferrara (Italy). All patients took part on a voluntary basis and were not remunerated for their participation. They were assured of the anonymity and confidentiality of the information provided and were informed that they could stop completing the questionnaire at any time if they so wished. They were also assured that the collected data would be used only for the purposes of the study.

## Author contributions

ST and JS contributed to conception and design of the study, planned the research project, and organized the database. JS wrote the first draft of the manuscript and was responsible for the review of the literature. ST contributed to the preparation of the manuscript and wrote sections of the manuscript. IC performed the statistical analysis. SC contributed to conception of the study, supervised the design of the study, and critically reviewed the manuscript. All authors contributed to manuscript revision, read, and approved the submitted version.

## Conflict of interest

The authors declare that the research was conducted in the absence of any commercial or financial relationships that could be construed as a potential conflict of interest.

## Publisher’s note

All claims expressed in this article are solely those of the authors and do not necessarily represent those of their affiliated organizations, or those of the publisher, the editors and the reviewers. Any product that may be evaluated in this article, or claim that may be made by its manufacturer, is not guaranteed or endorsed by the publisher.
